# Perceived Control and ICD Concerns in Older ICD Recipients: Sex as a Moderator

**DOI:** 10.1093/geroni/igab046.1892

**Published:** 2021-12-17

**Authors:** Abigail Latimer, Jennifer Miller, Misook Lee Chung, Muna Hammash, Debra Moser

**Affiliations:** 1 University of Kentucky College of Nursing RICH Heart Program, Lexington, Kentucky, United States; 2 University of Kentucky College of Nursing, Lexington, Kentucky, United States; 3 University of Louisville, Louisville, Kentucky, United States

## Abstract

Implantable cardioverter defibrillators (ICDs) reduce the risk of sudden cardiac death for those with a history of or high risk for lethal arrhythmias. In 2016, 105,000 ICDs were implanted in older adults (age ≥ 60) in the US. Approximately 25% of ICD recipients report significant ICD concerns with women reporting higher levels than men. Little is known about the experience of older adults living with life-saving/extending medical technologies, particularly related to sex differences in ICD concerns. Perceived control may decrease ICD concerns, but sex differences have not been explored. The aim of this cross-sectional study was to determine the moderation effect of sex on the association between perceived control and ICD concerns in older adults (age≥ 60). Participants completed a questionnaire on ICD Concerns (ICDC-8) and the Control Attitudes Scale-Revised, a measure of perceived control. We conducted a moderation analysis using Hayes’ PROCESS for SPSS with 5,000 bootstrap samples. Of the 99 participants (73.7% male; age 70 + 7 years; education 13 + 3 years), most participants were white (79.8%) and married (69.7%). There were no differences in participant characteristics, perceived control, or ICD concerns, by sex. We found an interaction between sex and perceived control (b= -.5715, p= 02), indicating that for women (-.5801, p =.007), as perceived control increased, ICD concerns decreased. For men (-.0089, p =.9439), ICD concerns remained the same despite level of perceived control. Future clinical and research interventions to decrease ICD concerns should include ways to increase perceived control particularly for older women living with ICDs.

